# An analysis of the quality of maternity services in Nampula, Mozambique: implementation research

**DOI:** 10.11604/pamj.2022.41.119.27301

**Published:** 2022-02-10

**Authors:** Paulo Pires, Martins Abudo Mupueleque, Jaibo Rassul Mucufo, David Zakus, Ronald Siemens, Celso Belo

**Affiliations:** 1 Faculty of Health Sciences, Lúrio University, Bairro de Marrere, Rua Nr. 4250, km 2.3, CP 364, Nampula, Mozambique,; 2 University Mussa Bin Bique, Nampula, Mozambique,; 3 Faculty of Health Sciences, Lúrio University, Nampula, Mozambique,; 4 Dalla Lana School of Public Health, University of Toronto, Toronto, Canada,; 5 Faculty of Medicine, University of Saskatchewan, Saskatoon, Canada,; 6 Faculty of Health Sciences, Lúrio University, Nampula, Mozambique

**Keywords:** Birth, delivery, health services, implementation research, maternal health, maternity, Mozambique, quality assessment

## Abstract

**Introduction:**

the quality of maternity services is an essential factor in reducing maternal and newborn morbidity and mortality, which remains extremely high in Africa. In Mozambique, maternal mortality rate is 451.6 deaths per 100,000 live births (2017). The reasons for this are complex, but one important factor to reduce this burden is to provide effective and efficient care, to improve institutional deliveries. To reduce maternal and newborn mortality rates in Nampula, researchers from Lúrio University and the University of Saskatchewan, carried out an implementation research program, including various interventions such as training activities for health professionals in maternal and child health care. We planned a mid-project evaluation, to assess the trainings´ impact on the quality of services at Marrere Hospital Maternity.

**Methods:**

quantitative pre-post study, carrying out two cross-sectional surveys about maternity service quality, one being conducted after five health professionals´ trainings and the other after six more trainings. The two surveys included samples of post-partum women in the maternity, calculated with a 10% margin error and 90% confidence interval for the first survey, and with a 7% margin error and 95% confidence interval for the second. The surveys were entered into REDCap and analysed to assess frequencies, percentages, mean and standard deviations. This research was approved by the Institutional Committees of Bioethics at Lúrio University and at the University of Saskatchewan.

**Results:**

one hundred and sixteen post-partum women were surveyed at the maternity, assessing standards of patient centred care during delivery labour. Most areas showed no improvement. Some positive improvements were delivering women were given the option to have a person of their choice accompany them during labour (75%), notably a traditional birth attendant (34%), and they had continuous support from a health professional (68%). But many shortcomings persisted in areas of privacy (33%) and confidentiality (57%).

**Conclusion:**

the quality of patient centred care at Marrere Hospital Maternity did not improve much with health professionals´ trainings. Decreasing the large turnover rate of such staff, reviewing their learning styles, and promoting continuous professional capacity building would be the next steps to improve quality of care.

## Introduction

Maternal and child mortality have their highest incidence around delivery time. Improving access to and quality of maternal and child health (MCH) services are essential to achieve sustainable development goals SDG3 and SDG5 [1], especially in developing countries; specifically reducing rates of morbidity and mortality in pregnant women and newborns, which in Mozambique are among the highest in Africa and the world. To reduce maternal and perinatal mortality associated with birth, labour management all over the world has been evolving, and there has been a shift to exclusive facility-based childbirth [2]. The World Health Organization (WHO) produced in 2018 specific recommendations for intrapartum care for a positive childbirth experience, to approach these issues [3].

In Mozambique, though the maternal and child mortality rates have been decreasing in the last two decades [4], are nevertheless still high (45.6 maternal deaths per 100,000 live births, 67.3 deaths of children less than one year of age per 1000 live births, 2017) [5]. Among the main causes are the lack of qualified health professionals (HPs), equipment and supplies, poor quality of care, deficient referral system, long distances to obtain services and lack of transport to access them at the appropriate health unit (HU), poor communication between HPs and the community, gender and traditional issues. These barriers to MCH, are common to low-and-middle income countries [6], mainly in sub-Saharan Africa [7]. Although the national Ministry of Health (MISAU) has defined policies to guarantee sexual and reproductive health (SRH) and rights in 2011 [8] in the last decade, the low quality of MCH care services in Mozambique has hardly improved [9]. An assessment of quality and access to health care, performed in 195 countries in 2016, placed Mozambique in position 179 (the 6^th^ worst) [10].

These facts led the Faculty of Health Sciences (FHS) at Lúrio University (UniLúrio) and the Department of Paediatrics at the University of Saskatchewan in Canada, to develop an implementation research on MCH, in Natikiri district, Nampula province, Mozambique, called Alert Community to a Prepared Hospital care continuum (ACPH). The baseline study, conducted at the initial stage with large community participation, showed a low level of knowledge about SRH and rights in the Natikiri population and poor family planning (FP) practice [11]. Project activities stimulated and enabled wide community participation and SRH and FP education, and also provided trainings for HPs in obstetric emergencies, newborn resuscitation, SRH rights, ante-natal consultation and humanization of care in Marrere General Hospital (MGH) and its associated health centre. One echograph and some other equipment and consumables were also provided to the maternity.

This paper pertains to the results of a planned mid-project evaluation, intended to estimate the impact of HPs trainings in MCH on quality of maternity services [12]. Given the importance of feedback from users to evaluate health services, with regard to the quality-of-care issues, communication, information, and advice, we surveyed user groups at the maternity following two HPs education interventions. This implementation research targeted participant´s citizenship and health empowerment, informing and educating population and HPs.

## Methods

**Design:** this was a quantitative pre-post study, applying two cross-sectional surveys on user´s opinion about maternity services quality at MGH, Natikiri district, Nampula, Mozambique. Post-partum women were randomly selected to survey in the MGH´ maternity, answering voluntarily a 30-minutes questionnaire, applied face to face by research assistants, unknown from participants.

**Activities:** the first survey was done during the 3^rd^ semester of the project (2018), after five training sessions (two on obstetric emergencies, two on newborn resuscitation and one on ante-natal consultation); the second survey was done on the 6^th^ semester (2019), after six more training sessions (one in ante-natal consultation, two more on newborn resuscitation, two on family-friendly consultation and humanized care, and one in SRH). Each training lasted five days (20 hours in total), given to 60HPs over the eleven modules, with an average number of 18 per session.

**Sample:** to calculate representative samples of post-partum women at MGH maternity, we considered the monthly average number of deliveries, 142 in 2018, applying a margin of error of 10% and a confidence interval of 90%, thereby attaining 47 women. And 166 in 2019; with a 95% confidence interval and a margin of error of 7%, attaining 91 women. The two groups are made up of different subjects, there were no repeated surveys.

**Data collection:** the research tool was a structured interview designed by the research team, following WHO recommendations [13, 14] and adopting MISAU recommendations of a “Maternity Model”. The questionnaire had 28 closed questions in Portuguese with multiple choice options, accessing principles of good care, communication with the woman, privacy and confidentiality, care throughout labour and birth, and three open questions: the best service, least pleasant, needs to do. This questionnaire was tested to evaluate validity and feasibility, with 10 post-partum women at the close by 25 *de Setembro* health centre´ maternity, and one adjustment was made adding “faces” expressing opinion (Annex 1).

The groups were surveyed in Portuguese or Emakhuwa (the local language) according to the participant´s preference. All were administered by UniLúrio FHS students, after being adequately trained and signing ethical and scientific commitment forms. Post-partum women were questioned in private at the MGH maternity, from 24^th^ to 31^st^ July 2018 and from 28^th^ November to 6^th^ December 2019. All women were informed they were free to participate voluntarily or abandon the survey if they wanted without any consequences in access or quality of care, received written information about research objectives and methods, risks and benefits, and signed an informed consent form, including an informed assent term for adolescents less than 18 years of age. There were no interviews refusals or abandons.

The questionnaires were answered using a 5-point Likert scale (i.e. totally agree, agree, indifferent, disagree, strongly disagree), were evaluated on the quality of completion by the principal investigator and introduced into Research Electronic Data Capture (REDCap) by the same students, accompanied by a FHS lecturer to consult as needed. The data were then analysed by a statistics professor to assess frequencies, percentages, means and standard deviations.

This study was approved by the Lúrio University´s Institutional Committee on Health Bioethics (02/CBISUL/16) and the Behavioural Ethics Board at the University of Saskatchewan (BEH#15-112) and followed all Helsinki Declaration (2013) guidelines.

## Results

We surveyed 116 post-partum women at MGH Maternity (24after five HPs trainings, 92 after six more), with a mean age of 23.6 years (standard deviation 5.7), minimum 14 and maximum 40 years (5.4% were less than 18 and 5.5% were more than 34). Concerning school level, 42.4% were illiterate, 44.6% had completed primary school and 12% secondary level, and one with higher education. Participants´ characteristics are detailed in Table 1.

**Table 1:** participant characteristics

			2018 (n=24)	2019 (n=92)	Progress (%)
No.	Question	Answer	Post 5 trainings	Post 11 trainings	
1	Residence (%)	Natikiri	83	97.8	18
		Other	17	2.2	-87
2	Number of previous pregnancies	Average (n)	2.6	3.1	19
		<= 3 (%)	71	68.5	-4
3	Number of hospital deliveries	Average (n)	2.4	2.45	2
4	Home births	(%)	12.5	24.2	94
5	Miscarriages	No (%)	83	82.4	-1
		Yes (%)	17	17.6	4

Legend: n - number of participants; % - percentage

Comparing 2018 with 2019, the proportions of residence locations changed, with an increase in Natikiri; there was a slight increase in previous numbers of pregnancies and maternity deliveries, but the reported percentage of past home deliveries increased two-fold; and there was no significant change in the percentage of women reporting miscarriage.

The assessment of principles of good care show a negative decline in all areas, including communication with patients, privacy and confidentiality, and care during labour and childcare: the patients felt less welcome at the maternity, HPs did not introduce themselves or asked if they had any doubts, HPs did not ask their name, did not encourage them to raise questions and state their expectations at the beginning of the consultation, and did not explain what they would do before performing physical examination or other interventions, did not encourage the husband´s participation in caring for the newborn (Annex 2).

Some positive points were identified, however, about care during labour: delivering women were given the option to have a person of their choice to accompany them during labour (75%), notably a traditional birth attendant (34%), they had continuous support from an HP (68%) and were able to deliver in a position of their choice (34%). The last question summarized the findings of participants´ perceptions, by asking the women how they evaluate their overall experience at the maternity: most women globally rated their experience in delivery as excellent (33%) and good (52%), but the evolution of this service, however, was unfavourable (Table 2).

**Table 2:** users' opinions about maternity service quality

		2018 (n = 24)	2019 (n = 92)	Progress (%)
Question	Response (%)	Post 5 trainings	Post 11 trainings	
How do you evaluate your experience delivering at Marrere General Hospital Maternity?	Great	25	33	-12
	Good	70.8	51.6	
	Not very satisfied	4.2	12.1	267
	Not satisfied	0	3.3	

Legend: N - number of participants; % - percentage

In answers to open questions in the 2019 survey, about what they liked in the maternity, 52 women (56.5%) liked reception, but 15 (16.3%) did not like anything. About what they did not like about the service, 12 women (13.6%) refer to HPs delays and 8 (9.1%) point to bad reception and treatment. About what they would change to make the service better, 56 (63.6%) would do nothing, 15 women (17.0%) reported improving the reception of patients by HP and 8 (9.1%) improving HPs punctuality.

## Discussion

The mean number of pregnancies per woman remains under the national average (5.2), with more than half having three or fewer pregnancies per woman, probably due to the low group mean age. Home births increased, or women felt more at ease revealing it, perhaps because of SRH education activities and population health empowerment. Miscarriages (spontaneous and provoked) show no change. This topic, is culturally sensitive, and we assume that the reality goes beyond the cases mentioned. HPs informed delivering women that they had the option to have a person of their choice to accompany them during labour, and this is a low-cost and effective intervention to improve the quality of maternity care [15], in a context where TBAs are pregnant women trusted partners.

In 2019, most post-partum women in MGH maternity are satisfied with the service, perhaps due to their low education level leading to lower expectations; but HPs did not generally proceed according to the rules of good care and the MISAU MCH protocol. They have deficiencies in patient reception, information, and communication, and in matters of confidentiality. In another study in Tanzania, mothers also reported mistreatment, failure to meet professional standards of care, poor interaction among women and providers [16]. HPs respectful and appropriate attitudes towards mothers, are essential to ensure the quality of childbirth experience; [17] but systematic reviews on maternity service quality, all over the world, have shown a widespread occurrence of different forms of mistreatment of women during delivery, impacting negatively on both clinical and psychological outcomes, and also poor staff knowledge and skills [18]. Our evaluation reveals no significant impact of HP trainings on maternity attendance quality. This finding might be related to the high turnover of MGH professionals, by a mandated reduction of MCH HPs, causing an overload of work to those remaining, and associated with a decrease in hospital economic resources, in parallel with low salaries and missing payments for extra-hours. On top of this, we verified 3 of the 11 training modules were not evaluated; in the 8 evaluated, we had 22% participants missing in the post-test; HPs had several training interruptions, due to hospital routine service tasks.

However, the number of MGH maternity deliveries show a positive quantitative evolution over time, from 1,243 in 2016 to 1,991 in 2019 (60% increase), high above the Mozambican population increase rate (2.8% average per year), despite the lack of MCH professionals. This could be an impact of other ACPH activities in the local community, through the training in MCH and SRHR of local health committees. Subsequent recommendations for MGH maternity professionals were directly transmitted verbally in follow-up meetings and written down and delivered to MGH Director and all HPs. We recommend a national birth attendants training campaign, continuous [19], and regular, on skills to deal with complications (breech birth, misrepresentation, multiple pregnancy and shoulder dystocia) [20], about values, transforming attitudes and interpersonal communication [21]. This must be combined with a significant improvement on maternity HPs working conditions.

**Study limitations:** first, as a potential study limitation, we point out the lack of the questionnaire psychometric testing. Second, location of interviews in MGH Maternity, that might have influenced some answers, due to the institutional environment. Another issue is the application of the Likert scale to a population with perceived difficulty in abstract conceptualization, in which the terms totally and partially may have been not well understood. Another limiting factor in comparing the two studies, is the use of a 90% confidence interval and 10% margin of error in the first sample, different from the second (95% and 7% respectively), as well as the inferior number of subjects in the first survey (24 of calculated 47) due to the low number of deliveries during the data collecting period. The limiting factor was the weak progress (17%) of mean evaluation of HPs trainings´ pre- and post-tests.

## Conclusion

Health systems today are faced with new (antimicrobial resistance, climate emergency, Covid-19 pandemic) and old (in Africa traditional healers´ preponderance, drugs interactions with medical plants) challenges, and will be forced to develop new intervention methods. MCH HPs are subjected to heavy workloads, especially in the maternity, and they do not usually practice according to protocol, having several shortcomings in patient´s reception, information, and communication. Although most post-partum women were satisfied with the care provided, and the maternity statistical indicators show improvement in the number of deliveries, we know maternal and child mortality incidence are the highest around delivery period. The Mozambican national health system continually faces challenges, looking for new tools for action. Continuous HP training and better staff working conditions are keys to achieve behaviour change and better maternity services quality. These interventions depend on MISAU being innovative and investment to: ensure the number required and continuity of MCH HPs at HUs; better HP working conditions; provide the necessary consumables for properly functioning services, including medicines, gloves, masks, and health information and education materials, and ensuring a user´s friendly environment at the HUs; promote recurrent trainings of MCH HPs, reinforcement and updating, in obstetric care, newborn care, humanized consultation, and patient centred and family friendly services.

### What is known about this topic

- Maternal and newborn mortality are high in Mozambique, and an important cause is low quality of health units' maternity services;
- Health professionals´ trainings improve quality of care services.

### What this study adds

- Health professionals training in maternal and child health is not sufficient to increase quality of maternity services;
- Health professionals need better working conditions, stability, and salaries to improve their productivity.

## Annex

**Annex 1**: research tool to collect users' opinions on Maternity services quality, to assess the principles of good care, at Marrere General Hospital, Natikiri, Mozambique

**Annex 2**: users' opinions on Maternity services quality, to assess the principles of good care, at Marrere General Hospital, Natikiri, Mozambique
